# Electrospun aligned tacrolimus-loaded polycaprolactone biomaterials for peripheral nerve repair

**DOI:** 10.2217/rme-2023-0151

**Published:** 2023-10-11

**Authors:** Holly N Gregory, Owein Guillemot-Legris, Daisy Crouch, Gareth Williams, James B Phillips

**Affiliations:** 1 UCL School of Pharmacy, University College London, London, WC1N 1AX, UK; 2 UCL Centre for Nerve Engineering, London, WC1N 1AX, UK

**Keywords:** alignment, electrospinning, nanofibers, nerve regeneration, neurons, PCL, Schwann cells, tacrolimus

## Abstract

**Background:** Efficacious repair of peripheral nerve injury is an unmet clinical need. The implantation of biomaterials containing neurotrophic drugs at the injury site could promote nerve regeneration and improve outcomes for patients. **Materials & methods:** Random and aligned electrospun poly-ε-caprolactone scaffolds containing encapsulated tacrolimus were fabricated, and the gene expression profile of Schwann cells (SCs) cultured on the surface was elucidated. On aligned fibers, the morphology of SCs and primary rat neurons was investigated. **Results:** Both scaffold types exhibited sustained release of drug, and the gene expression of SCs was modulated by both nanofibrous topography and the presence of tacrolimus. Aligned fibers promoted the alignment of SCs and orientated outgrowth from neurons. **Conclusion:** Electrospun PCL scaffolds with tacrolimus hold promise for the repair of peripheral nerve injury.

## Plain language summary

This article reports the production and testing of fibrous materials loaded with tacrolimus, a drug known to improve nerve regeneration, for the surgical repair of peripheral nerve injury. Materials were created with either a randomly orientated structure or an aligned structure that mimics the anatomy of native nerve, and both displayed long-term release of the loaded drug. Schwann cells, which are a critical cell type in nerve regeneration, were grown on the materials and their behaviour was positively influenced by the fibrous surfaces and/or the presence of tacrolimus. Neurons grown on the aligned materials demonstrated directional outgrowth, which may be also beneficial for increasing the rate of regeneration. These materials have the potential to improve outcomes of nerve repair for patients.

Tweetable abstract

Aligned tacrolimus-loaded polycaprolactone biomaterials for peripheral nerve repair modulate Schwann cell gene expression and promote directional outgrowth from primary neurons.

Peripheral nerve injury affects 11 people out of every 100,000 in the UK per year [1], and often requires surgical intervention to promote the return of sensory or motor function. In a transection injury scenario this procedure unites the two ends, and when a large gap in the nerve tissue is evident it usually involves implanting an autologous nerve graft as a bridge between the stumps. However, regeneration after these procedures is slow and patients continue to suffer neuropathic pain alongside socioeconomic consequences and post-traumatic stress [2,3]. A promising avenue in preclinical research is the development of neurotrophic drug-eluting biomaterials that could be implanted at the site of injury during surgical repair, in order to bolster regeneration and so hasten the return of function (discussed in [4]).

Tacrolimus, or FK506, is a macrolide that is widely used to prevent rejection in hand and renal transplant patients. In addition to its immunosuppressant properties, tacrolimus has been known as a potent driver of nerve regeneration for many years both *in vitro* [5,6] and in animal models [7–9]. In humans, its clinical use in hand transplantation and arm replantation [10–12] has been credited with rapid axonal regrowth. The mechanism of this neurotrophic action is unknown but has been linked to its binding to FK506-binding protein 52 and subsequent activation of the ERK pathway, in addition to preventing inactivation of GAP-43 and TGFβ-1 via FK506-binding protein 52 (reviewed in [13–15]). However, alongside immunosuppression, systemic delivery of tacrolimus has been associated with brain and kidney toxicity, infection, gastrointestinal disturbance and diabetogenesis [16]. A treatment approach involving local delivery of tacrolimus at the repair site, using biomaterials as a drug depot, may provide a sufficient dose to promote nerve growth while avoiding side effects from systemic administration [17–20].

Electrospinning is a scalable fabrication technique that produces polymeric fibers from nanoscale to microscale that can be collected to form fibrous scaffolds. This process can create materials with structural similarities to native extracellular matrix [21], can incorporate regenerative agents such as small-molecule drugs, and can generate topographically aligned materials – as such, it has been used extensively to create nerve constructs preclinically [22]. Aligned poly-ε-caprolactone (PCL) fibers in particular have been demonstrated to direct [23–27] and enhance [28] outgrowth of primary neurons seeded on their surface, and to promote the orientation and/or elongation [29,30] and migration rate [31] of Schwann cells – these effects may be beneficial for nerve regeneration. Indeed, aligned fibers encourage more robust regeneration compared with random fibers in the repair of rat models of nerve injury [32–34] and polarise macrophages towards a pro-healing phenotype alongside promoting Schwann cell infiltration [35]. As such, the combination of aligned PCL fibers with the delivery of a pro-regenerative small molecule may be a valuable development in preclinical nerve repair research.

In this study, we fabricated random and aligned tacrolimus-loaded PCL scaffolds by electrospinning and investigated their impact on the gene expression profile of Schwann cells cultured on their surface in order to measure their regenerative potential. Furthermore, we evaluated the effect of the aligned fibers on Schwann cell morphology, and examined how fiber orientation and tacrolimus content influenced the rate and directionality of outgrowth from primary rat neurons.

## Materials & methods

### Electrospinning

Electrospinning was conducted on a Fluidnatek LE-50 (Bioinicia, Spain). For randomly oriented fibers, a static coaxial spinneret with inner/outer needle internal diameters of 0.6/1.4 mm was positioned 10 cm from a 25 mm-diameter mandrel, rotating at 200–400 RPM and supplied with a -2.0 kV voltage. Solutions of 15% w/v 80 kDa PCL in 90/10 v/v trifluoroethanol/dH_2_O (shell) and 10 mg/ml tacrolimus in ethanol (core) were fed to the spinneret at 1000 and 200 μl/h, respectively, for a theoretical loading of 1.3% w/w. Fibers were collected under a voltage of 10.5 kV, at 24.5 ± 1.7°C and 39 ± 8% humidity, then stored at -20°C. Random blank PCL fibers were generated by electrospinning of the 15% PCL solution under identical collection conditions. For aligned fibers, a static monoaxial spinneret with a needle internal diameter of 1.4 mm was positioned 20 cm from a 100 mm-diameter mandrel, rotating at 1750 RPM and supplied with a -2.0 kV voltage. A solution of 12.5% w/v 80 kDa PCL in trifluoroethanol with 2 mg/ml tacrolimus was fed to the spinneret at 750 μl/h, for a theoretical loading of 1.6% w/w. Fibers were collected under a voltage of 7.5–8.0 kV, at 24.9 ± 1.5°C and 34 ± 6% humidity, then stored at -20°C. Aligned and random blank PCL fibers were generated by electrospinning of the 12.5% PCL solution under identical collection conditions.

### Scanning electron microscopy

Fiber morphology was assessed by sputter coating fibers mounted on a carbon tab with gold using a Q150R coater (Quorum, UK) and imaging on either a field emission Quanta 200 scanning electron microscope (SEM; FEI, UK) with a secondary electron detector or a Phenom™ Pro G6 Desktop SEM (Thermo Fisher Scientific, MA, USA). Fiber diameter measurements were performed on ImageJ software [36] – at least 130 points of measurement were taken across nine individual SEM images acquired from three separate fiber mats. Fiber alignment was determined using the ImageJ Directionality plugin, using nine SEM images across three separate fiber mats, and outputs were normalized with the highest frequency at 0° for facile comparison across images.

### x-ray diffraction

x-ray diffraction (XRD) patterns of fiber and drug samples were collected on a benchtop Miniflex 600 x-ray diffractometer (Rigaku, Japan), at a wavelength of 1.5418 Å, over a range of 3–50° at 5°/min with a step of 0.020°, an accelerating voltage of 40 kV and a current of 15 mA.

### Fourier-transform infrared spectroscopy

Attenuated total reflectance-Fourier transform infrared spectroscopy (FTIR) was performed on a Spectrum 100 FTIR spectrometer (PerkinElmer, MA, USA) using 16 separate scans and 4 cm^-1^ resolution over the range 650–4000 cm^-1^. For XRD and FTIR of drug-loaded fibers, three samples from three separate fiber mats were tested in each analysis.

### Tacrolimus release & detection

To assess release kinetics, 10.0 ± 0.1 mg of random tacrolimus fibers (n = 3 separate fiber mats, three sections per mat) and 10.0 ± 0.2 mg of aligned tacrolimus fibers (n = 3 separate fiber mats, one section per mat) were incubated with 1 ml release buffer (50 mM NH_4_HCO_3_ ± 0.02% NaN_3_) in microtubes in an Incu-shake MINI shaking incubator (SciQuip, UK) set at 37°C and 75 RPM. Release buffer was exchanged every 2–3 days and then stored at -20°C prior to determination of tacrolimus content. Liquid chromatography-mass spectrometry was used to detect tacrolimus released from fibers, as reported previously [37]. Briefly, a 1260 Infinity liquid chromatography system (Agilent, UK) coupled to a 6460 triple quadrupole mass spectrometer (Agilent) was used with an Agilent Pursuit 5 μm C8 50 × 2.1 mm column and gradient elution of water and methanol mobile phases, buffered with 10 mM ammonium formate and 0.05% formic acid. A validated protocol was used as the basis for analysis [38].

### Cell culture

#### SCL 4.1/F7 Schwann cell line

The SCL 4.1/F7 (ECACC 93031204) Schwann cell line derived from neonatal Wistar rats was obtained from the European Collection of Authenticated Cell Cultures. Cells were maintained below 70% confluency in high-glucose Dulbecco’s Modified Eagle’s Medium (DMEM) supplemented with 10% fetal bovine serum (FBS) and 100 U/ml penicillin and 100 μg/ml streptomycin (P/S), and were passaged using 0.25% trypsin/EDTA for detachment from the culture surface. Schwann cells were used in experiments up to passage 20.

### Seeding of Schwann cells on random & aligned fibers

Disks of random and aligned tacrolimus-loaded fibers and respective blank materials approximately 15 mm in diameter were sterilized by UV for 30 min, then immersed in FBS for 1 h at room temperature. For PCR and LDH assay, Schwann cells were seeded onto random and aligned fibers and onto FBS-coated tissue culture plastic (TCP) at 50,000–60,000 cells/well. For immunocytochemistry, Schwann cells were seeded at 25,000 cells/well. All conditions were then incubated for 48 h at 37°C, and for immunocytochemistry studies the cells were fixed in 4% paraformaldehyde for 30 min at room temperature. Schwann cell experiments were performed in triplicate (n = 3 fiber disks per condition), using separate drug-loaded fiber mats and different cultures of Schwann cells in each experiment.

### LDH assay

After 48 h incubation, cells in dead control wells were exposed to 1% triton-X100 for 1 h and thereafter all media was removed and immediately analysed for lactate dehydrogenase activity using a commercial kit (Abcam). Briefly, 10 μl of culture media was combined with 100 μl of 1:50 water-soluble tetrazolium salt:assay buffer and the mixture incubated at 37°C. The absorbance was read at 450 nm and 650 nm and the latter value subtracted during analysis. Values were normalized to the dead control groups.

### RNA extraction & qPCR

Buffer RLT Plus was used to immerse fibers with cultured cells and those adhered to TCP, then all materials were immediately frozen at -80°C. Total RNA was extracted using a RNeasy Plus Mini Kit according to the manufacturer’s instructions (Qiagen). cDNA was synthesized using a GoScript Reverse Transcriptase kit (Promega). Real time-quantitative PCR (RT-qPCR) was performed with a QuantStudio 3 instrument (Applied Biosystems, UK) and analysed with QuantStudio Design & Analysis Software (Applied Biosystems). PCR reactions were run using a Power SYBR Green PCR Master Mix. The products were analysed by performing a melting curve at the end of the PCR. Data were normalized to the mRNA expression of three reference genes: *B2m*, *HPRT1* and *RPS18*. Primer sequences for qPCR are listed in Table 1.

**Table 1.** Primer sequences for qPCR.

Name	Symbol	Forward primer (5′–3′)
		Reverse primer (5′–3′)
Brain-derived neurotrophic factor	*Bdnf*	AATGCCGAACTACCCAATC
		CATACACAGGAAGTGTCTATCC
Beta-2 microglobulin	*B2m*	CGTGATCTTTCTGGTGCTTG
		GGTGGAACTGAGACACGTAG
C-C motif chemokine ligand 2	*Ccl2*	GCAAGATGATCCCAATGAGTC
		GCTTGGTGACAAATACTACAGC
Ciliary neurotrophic factor	*Cntf*	CAGACCTGACTGCTCTTATGG
		TGCTTGCCACTGGTACAC
Hypoxanthine phosphoribosyltransferase 1	*Hprt1*	ACCTCTCGAAGTGTTGGATAC
		GATTCAAATCCCTGAAGTGCTC
Interleukin-6	*Il6*	GGAGACTTCACAGAGGATACC
		CAGAATTGCCATTGCACAAC
Ribosomal protein S18	*Rps18*	CTTCGCTATCACTGCCATTAAG
		GTGAGGTCAATGTCTGCTTTC
Glial cell line-derived neurotrophic factor	*Gdnf*	GCTGACCAGTGACTCCAATATG
		TGCCGCCGCTTGTTTATC
Activating transcription factor 3	*Atf3*	GAGATTCGCCATCCAGAAC
		CAGACTTGGTGACTGACATC
Jun proto-oncogene, AP-1 transcription factor subunit	*Jun*	GACTGCAAAGATGGAAACGAC
		AGGGTTACTGTAGCCGTAGG
Leukaemia inhibitory factor	*Lif*	CAAGAGTCAACTGGCTCAAC
		GCATGGAAAGGTGGGAAATC
SRY-box transcription factor 10	*Sox10*	CCACATCGACTTCGGCAATG
		CCAGCTCAGTCACATCAAAGG

### Dorsal root ganglia dissection & culture

An adult male Sprague–Dawley rat was culled by CO_2_ asphyxiation and dorsal root ganglia (DRGs) were dissected from the spinal column then incubated for 90 min in 0.125% collagenase type IV in DMEM supplemented with P/S. DRGs were then dissociated by mechanical trituration and spin washed twice at 200 *× g* in DMEM supplemented with 10% FBS and P/S. The suspension was seeded into a poly-d-lysine coated flask supplemented with 0.02 mM cytosine arabinofuranoside, then incubated for 24 h before being detached using 0.25% trypsin/EDTA.

### Seeding of primary neurons on random & aligned fibers

Disks of aligned tacrolimus-loaded fibers in addition to aligned and random blank fibers 15 mm in diameter were sterilized by UV for 30 min, then immersed in FBS for 1 h at room temperature. Primary neurons were resuspended in neurobasal A medium supplemented with 1% B27, 2 mM l-glutamine and P/S, then seeded onto fibers at 10,000 cells/well. Neurons were incubated for 72 h at 37°C, then fixed in 4% paraformaldehyde for 30 min at room temperature. Neuronal morphology experiments were conducted using DRGs from one rat (n = 3 fiber disks per condition), to avoid the unnecessary use of animal tissue as initial results indicated that further optimization of tacrolimus dosage was required.

### Immunostaining & imaging

Schwann cells cultured on electrospun fibers were stained for F-actin and primary neurons were immunostained for βIII-tubulin. Fiber samples were first immersed in 0.1% Sudan black B in 70% ethanol for 40 min to reduce autofluorescence, and then cells were permeabilized with 0.5% Triton-X100 in PBS for 20 min. Schwann cells were treated with 1:200 rhodamine-conjugated phalloidin (00032, Biotium) in PBS overnight at 4°C. Neurons were blocked with 5% goat serum in PBS, treated with 1:400 rabbit α-βIII-tubulin (T2200, Merck) in 5% goat serum in PBS overnight at 4°C, then incubated with 1:200 DyLight goat α-rabbit 488 (DI-1488, Vector Laboratories, UK) in 5% goat serum in PBS for 45 min at room temperature. All nuclei were stained using Hoechst 33342 1:1000 in PBS for 10 min. Primary neurons were imaged on a Zeiss LSM 980 with Airyscan 2 by 4 × 4 tile scanning at ×20, with one tile scan per fiber disk. Schwann cells were imaged on a Zeiss Axio Lab. A1 at ×10, with five fields captured per fiber disk.

### Image analysis

The orientation of Schwann cells was assessed using Volocity 6.5.1., where the angle of the thirty longest cells per field in each of the five fields was determined. The mean angle for each image was calculated, and then the deviation of each cell from this mean was ascertained. The orientation and length of primary neurons was measured using Volocity with a similar protocol, where the length and angle of the twenty longest cells was quantified then the deviation of each angle from the mean of the image was obtained.

### Statistics

All data were assessed for normality by Kolmogorov-Smirnov test and where appropriate significance was assessed by one-way ANOVA with Tukey’s *post hoc* test using GraphPad Prism 9.4.1., where *p < 0.05, **p < 0.01, ***p < 0.001 and ****p < 0.0001.

## Results

**Figure 1.**
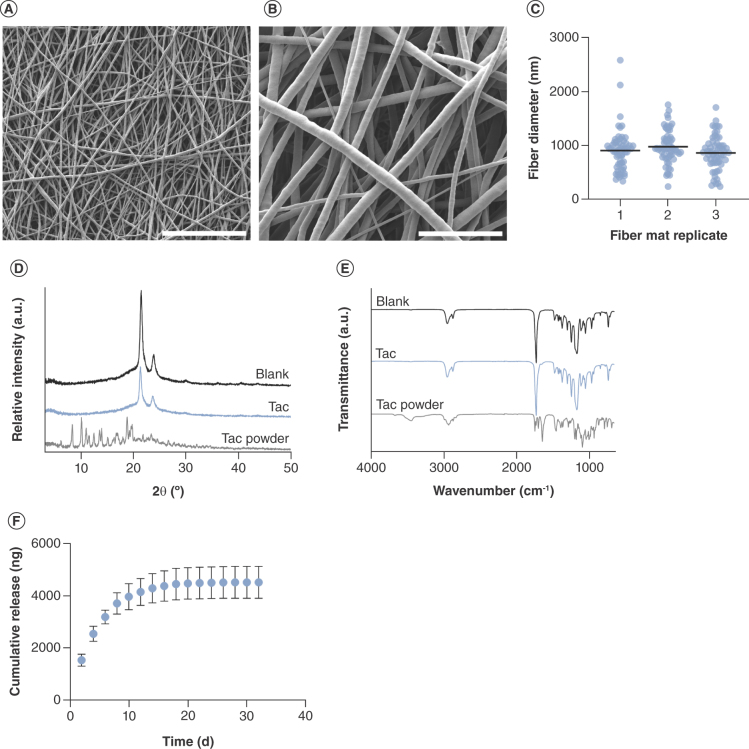
Characterization of randomly orientated tacrolimus-loaded fibers. Scanning electron microscope images of random tacrolimus-loaded poly-ε-caprolactone fibers, scale bar = 50 μm **(A)** and 10 μm **(B)**. **(C)** Fiber diameter of three separate mats, mean ± SD. **(D)** x-ray diffraction plots and **(E)** FTIR spectra compared with blank fibers and tacrolimus powder. **(F)** Cumulative release of tacrolimus from nanofibers, mean of n = 3 ± SD. Blank: Poly-ε-caprolactone fibers; Tac: Tacrolimus-loaded poly-ε-caprolactone fibers.

Coaxial electrospinning of tacrolimus and PCL at a low mandrel speed of 200–400 RPM generated a randomly orientated material (Figure 1A) comprised of smooth cylindrical fibers (Figure 1B) with a mean diameter of 917 ± 339 nm (Figure 1C). Characterization by XRD revealed strong reflections at 2θ = 21.4° and 23.8° (21.5° and 23.9° for blank fibers), which are consistent with the semi-crystalline nature of the polymer and correlate to the (110) and (200) crystal planes of the PCL orthorhombic form (Figure 1D) [39]. Reflections indicative of tacrolimus were not present in the fibers. FTIR spectra of the fibers were also collected, and those containing encapsulated tacrolimus were similar to the blank fibers, including a doublet around 3000 cm^-1^, which is characteristic of the asymmetric and symmetric C-H stretching in the PCL methylene groups and a strong peak at 1722 cm^-1^ for the ester carbonyl group (Figure 1E) [40]. Notably, the drug-loaded fiber spectrum does not display the broad O-H stretching peak around 3400 cm^-1^ or the C = C stretching peak at 1637 cm^-1^ exhibited in the tacrolimus spectrum. Release of tacrolimus from the fibers was assessed and was sustained over 32 days, with approximately 4.5 μg of drug eluted from 10 mg of material in this timeframe (Figure 1F). Coaxial electrospinning produced uniform tacrolimus-loaded fibrous scaffolds with consistent diameter and controlled drug release over multiple weeks.

**Figure 2.**
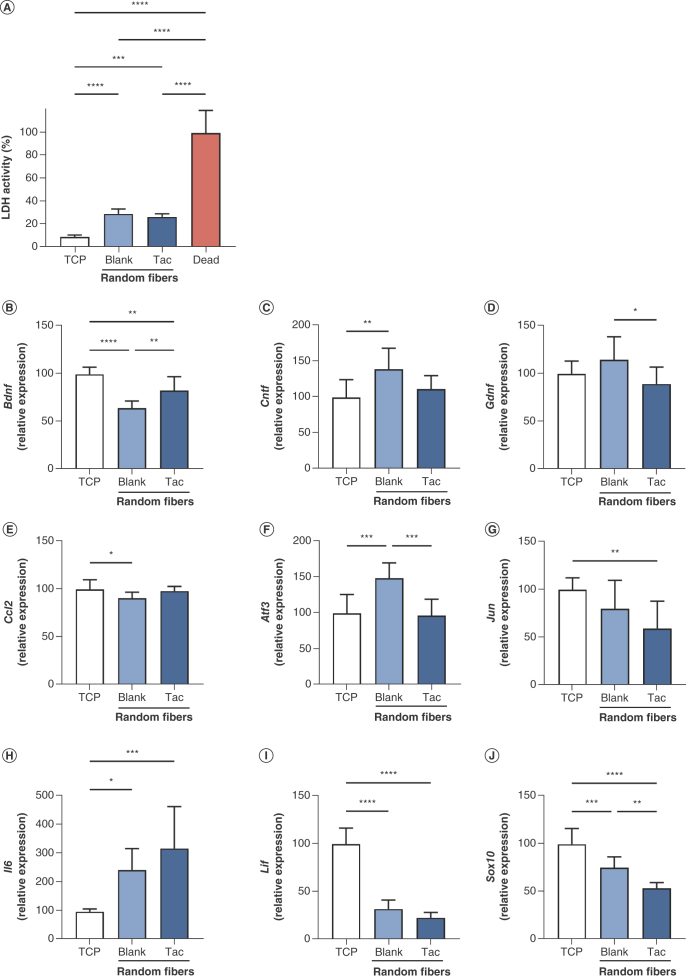
Survival and differential gene expression of Schwann cells cultured on randomly orientated tacrolimus-loaded and blank poly-ε-caprolactone fibers at 48 h. **(A)** LDH activity in culture media of cells grown on tissue culture plastic (TCP) and electrospun fibers, normalized to dead control group, mean ± SD. Relative expression of **(B)***Bdnf*, **(C)***Cntf*, **(D)***Gdnf*, **(E)***Ccl2*, **(F)***Atf3*, **(G)***Jun*, **(H)***Il6*, **(I)***Lif* and **(J)***Sox10*, normalized to TCP group, mean of n = 3 in triplicate ± SD. One-way ANOVA with Tukey’s *post hoc* test. *p < 0.05; **p < 0.01; ***p < 0.001; ****p < 0.0001; otherwise nonsignificant.

To investigate the feasibility of the drug-loaded fibers as a scaffold for regeneration in nerve repair, Schwann cells were cultured on the randomly orientated materials and cell death assessed after 48 h using a lactate dehydrogenase (LDH)-based assay (Figure 2A). Data were normalized to the dead control, and all conditions had at least threefold lower LDH activity compared with this group (p < 0.0001). LDH activity in the media of cells cultured on blank and tacrolimus-loaded fibers was similar (p > 0.05), and approximately threefold higher (p < 0.0001 and p < 0.001, respectively) than that of the TCP group. The majority of seeded Schwann cells appeared to survive on the electrospun fiber surfaces, and the presence of tacrolimus did not impact viability of cultured cells.

RT-qPCR was used to determine any changes in the expression of nine selected genes in Schwann cells when cultured on tacrolimus-loaded materials compared with those on TCP and blank fibers (Figure 2B–J). Four genes from this panel were differentially expressed in drug-loaded fibers compared with blank, demonstrating the effect of tacrolimus independently of the nanofibrous surface topography. Notably, *Bdnf*, a neurotrophin secreted in the CNS and PNS, was significantly upregulated in Schwann cells cultured on tacrolimus-loaded fibers (p < 0.01). The remaining genes exhibited reduced expression on drug-loaded materials, including *Gdnf*, a neurotrophic factor upregulated by Schwann cells in response to injury (p < 0.05) [41], *Atf3*, a factor induced by the cellular stress response (p < 0.001) [42] and *Sox10*, a transcription factor crucial for the development and maintenance of Schwann cells (p < 0.01) [43].

The nanofibrous topography of the randomly orientated materials also appeared to modulate gene expression, separately to drug exposure. Compared with TCP, Schwann cells cultured on blank fibers significantly downregulated the expression of *Bdnf* (p < 0.0001), *Ccl2*, a chemokine which regulates the movement of monocytes and macrophages (p < 0.05) [44], *Lif*, a cytokine involved in inflammation (p < 0.0001) [45], and *Sox10* (p < 0.001). The latter was also reduced further in the tacrolimus-loaded group (p < 0.01). Conversely, *Cntf*, a cytokine known in part as a survival factor for neuronal cell types (p < 0.01) [46,47], *Atf3* (p < 0.001) and *Il6*, a mediator molecule in inflammatory and immune reactions (p < 0.05) [48], displayed significant upregulation on blank nanofibers. The latter demonstrated the greatest differential in expression of all groups, where cells on tacrolimus-loaded fibers upregulated *Il6* by more than 300% compared with those on TCP (p < 0.001). Finally, the expression of *Jun*, a transcription factor activated in Schwann cells after injury that globally dictates their phenotypic shift into specialized repair cells [49], was significantly decreased in the drug-loaded fiber group compared with TCP (p < 0.01). Gene expression in Schwann cells cultured on the materials was markedly influenced by nanofibrous topography, which resulted in downregulation of factors including *Bdnf* and *Sox10* alongside upregulation of *Il6*. It was also influenced by exposure to tacrolimus, which increased expression of *Bdnf* while reducing that of *Sox10* and *Gdnf*.

**Figure 3.**
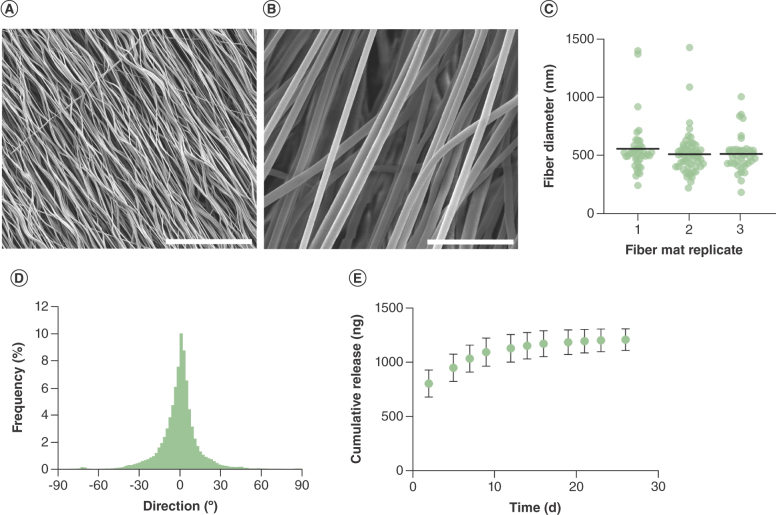
Characterization of aligned tacrolimus-loaded fibers. Scanning electron microscope images of aligned tacrolimus-loaded poly-ε-caprolactone fibers, scale bar = 50 μm **(A)** and 5 μm **(B)**. **(C)** Fiber diameter of three separate mats, line = mean. **(D)** Directionality histogram of alignment. **(E)** Cumulative release of tacrolimus from nanofibers, mean of n = 3 ± SD.

Blend electrospinning of tacrolimus and PCL onto a rapidly rotating mandrel generated highly aligned and uniform fibers (Figure 3A & B) with a mean diameter of 526 ± 192 nm (Figure 3C). Fiber directionality was quantified and confirmed that the materials were orientated, where 52% of the fibers lay within 15° of the predominant direction (Figure 3D). Tacrolimus was eluted from the aligned fibers over 26 days, with 1.2 μg of drug detected across the duration (Figure 3E). The aligned fibers exhibited a more pronounced burst release effect than randomly orientated materials, alongside a period of drug elution that was 6 days shorter and an almost fourfold lower magnitude of total drug detected despite theoretically similar loading during fabrication.

**Figure 4.**
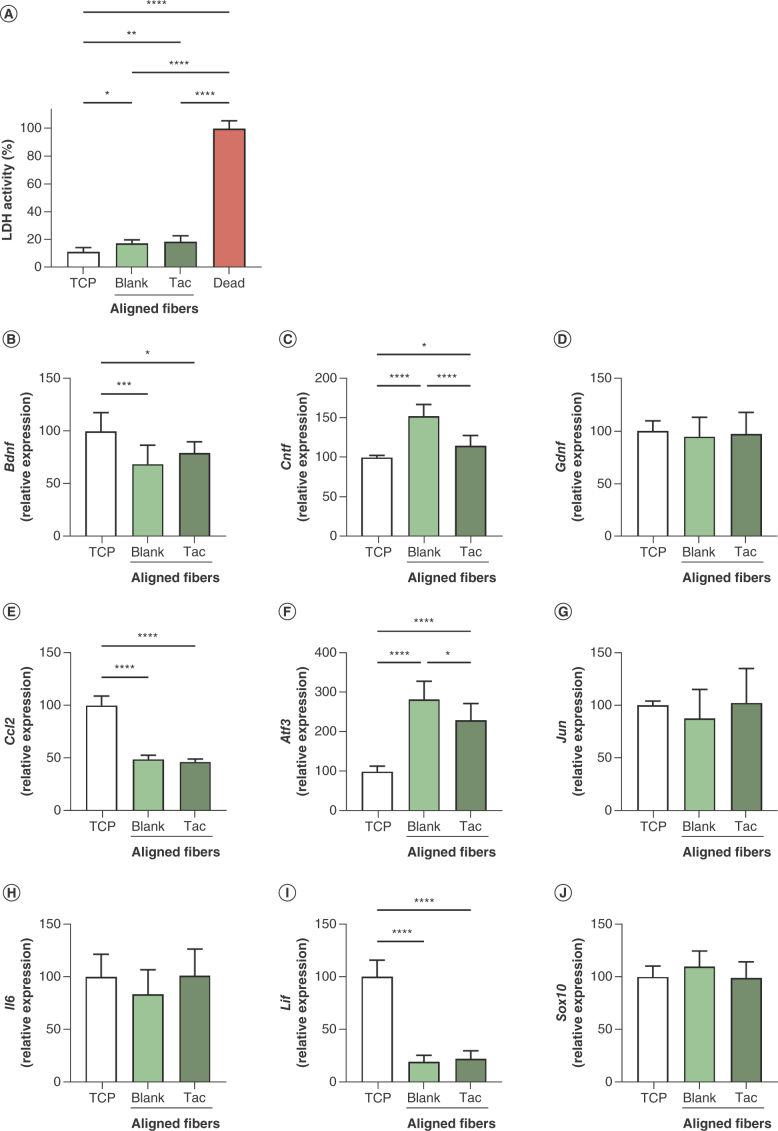
Survival and differential gene expression of Schwann cells cultured on aligned tacrolimus-loaded and blank poly-ε-caprolactone fibers at 48 h. LDH activity in culture media of cells grown on tissue culture plastic (TCP) and electrospun fibers, normalized to dead control group, mean ± SD **(A).** Relative expression of **(B)***Bdnf*, **(C)***Cntf*, **(D)***Gdnf*, **(E)***Ccl2*, **(F)***Atf3*, **(G)***Jun*, **(H)***Il6*, **(I)***Lif* and **(J)***Sox10*, normalized to TCP group, mean of n = 3 in triplicate ± SD. One-way ANOVA with Tukey’s *post hoc* test. *p < 0.05; **p < 0.01; ***p < 0.001; ****p < 0.0001; otherwise nonsignificant.

Schwann cells were also cultured on aligned tacrolimus-loaded fibers and LDH activity used to assess relative cell death after 48 h (Figure 4A). All experimental groups displayed at least 80% less LDH activity than the dead control (p < 0.0001). As for randomly orientated materials, the blank and tacrolimus-loaded fiber groups were similar for aligned materials (p > 0.05), and were 1.5 (p < 0.05) and 1.7-fold (p < 0.01) higher than the TCP condition. Schwann cells exhibited a low proportion of cell death when cultured on aligned electrospun fibers, and as for random fibers any exposure to tacrolimus associated with the scaffolds did not affect cell viability.

RT-qPCR was again used to determine whether expression of the nine selected genes was altered when cultured on the aligned drug-loaded fibers, compared with aligned blank materials and TCP (Figure 4B–J). Two genes from this panel displayed differential regulation in tacrolimus-loaded fibers compared with blank: the expression of *Cntf* was reduced by 25% (p < 0.0001), and that of *Atf3* by almost 20% (p < 0.05). Notably, the latter was also reduced between these groups on random materials, although *Cntf* expression was not significantly different previously. The changes in expression of neurotrophic factors *Bdnf* and *Gdnf* alongside transcription factor *Sox10* found between these groups on random fibers was also not exhibited on aligned fibers.

Independently of the effect of tacrolimus, the aligned nanofibrous topography also affected the expression profile of Schwann cells cultured on the surface compared with TCP. As on blank random fibers, *Bdnf* (p < 0.001), *Ccl2* (p < 0.0001) and *Lif* (p < 0.0001) were significantly downregulated on blank aligned fibers versus TCP, and *Ctnf* (p < 0.0001) and *Atf3* (p < 0.0001) were upregulated. In the majority of these factors, the magnitude of these changes was greater on aligned fibers – for example, *Ccl2* expression on TCP was reduced by 9% on blank random fibers and by over 50% on blank aligned fibers. Similarly, *Atf3* expression compared with the respective TCP group was 150% on random fibers and 280% on aligned fibers. Interestingly, *Il6* and *Sox10* expression on drug-loaded and blank-orientated materials was unchanged compared with TCP, which is unlike the significant effects observed for these genes on random materials. Expression of *Gdnf* and *Jun* was also similar across the TCP, blank aligned and tacrolimus-loaded aligned fiber conditions, whereas in random fibers these factors were modulated by either tacrolimus exposure or the combination of topography and tacrolimus presence. Overall, on the aligned fibers Schwann cell gene expression was influenced more by the change of culture surface from TCP to an aligned nanofibrous topography than by the presence of tacrolimus. In addition, fewer genes were differentially expressed on aligned materials than on random materials, both in terms of TCP compared with blank fibers and blank fibers in comparison to drug-loaded fibers.

**Figure 5.**
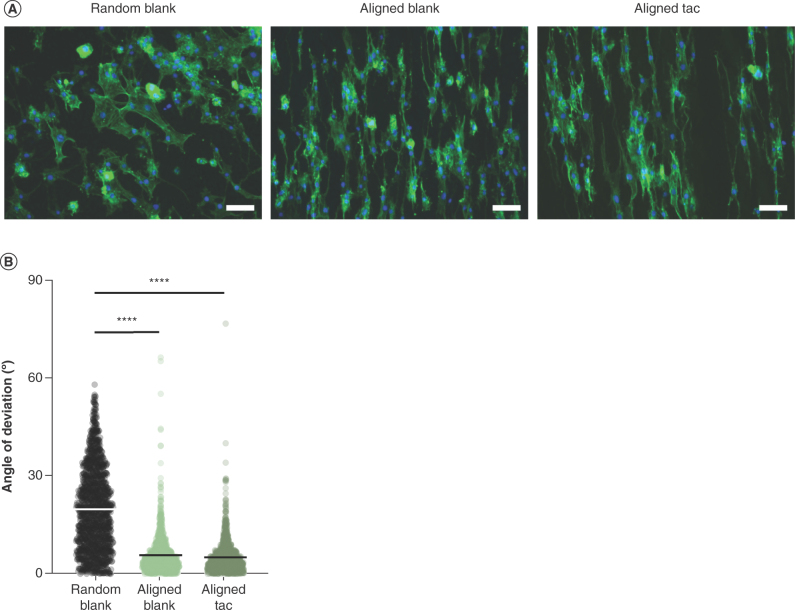
Orientation of Schwann cells on aligned tacrolimus-loaded and blank poly-ε-caprolactone fibers. **(A)** Fluorescence micrographs of Schwann cells cultured on random blank fibers, aligned blank fibers and aligned tacrolimus-loaded fibers, F-actin stained with phalloidin (green) and nuclei stained with Hoechst (blue), scale bar = 100 μm. **(B)** Angle of deviation of Schwann cells from the mean of each image, line = mean of n = 3 in triplicate. One-way ANOVA with Tukey’s *post hoc* test. ****p < 0.0001; otherwise nonsignificant.

To investigate the impact of aligned nanofibrous topography on cell morphology, Schwann cells were grown on aligned blank and tacrolimus-loaded fibers in addition to randomly orientated blank materials (Figure 5A & B). Schwann cells appeared to adopt an orientated and elongated morphology on both sets of aligned fibers, being comparatively more spread with no clear directionality on random fibers (Figure 5A). The orientation of the cells was quantified, which revealed that the Schwann cells deviated significantly more (p < 0.0001) when cultured on random materials than on either blank or tacrolimus-loaded aligned materials, and that there was no significant effect of the drug on angle of deviation (Figure 5B).

**Figure 6.**
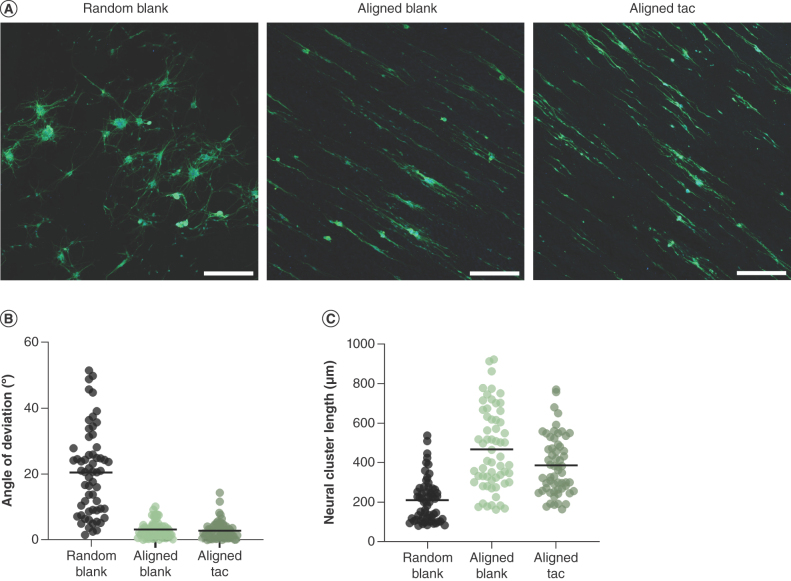
Orientation and outgrowth of primary rat neurons on aligned tacrolimus-loaded and blank poly-ε-caprolactone fibers. **(A)** Fluorescence micrographs of neural clusters of primary rat neurons cultured on random blank fibers, aligned blank fibers and aligned tacrolimus-loaded fibers, immunostained for βIII-tubulin (green) and nuclei stained with Hoechst (blue), scale bar = 300 μm. **(B)** Angle of deviation of clusters from the mean of each image, line = mean and **(C)** length of neural clusters, line = mean.

Primary neurons from rat dorsal root ganglia were cultured on the surface of the aligned drug-loaded scaffolds to further investigate their potential as biomaterials for peripheral nerve repair, and cell morphology was assessed in comparison to aligned and random materials without drug (Figure 6A–C). Neurons attached to all nanofibrous materials in culture and formed clusters, and on aligned blank and tacrolimus-loaded fibers these structures appeared to extend neurites directionally (Figure 6A). The orientation of these clusters was quantified, and those on both sets of aligned materials displayed considerably less deviation than on random fibers (Figure 6B). The length of these clusters was also evaluated, which indicated that those on aligned fibers were longer than on random, and that blank fibers appeared to incite slightly more outgrowth than tacrolimus-loaded materials (Figure 6C).

## Discussion

This work developed formulations of random and aligned tacrolimus-loaded electrospun PCL fibers as biomaterials for the repair of peripheral nerve injury. The encapsulation of tacrolimus into electrospun scaffolds for nerve repair has been reported previously, using PCL [37], polycarbonate-urethane [17], and polyvinyl alcohol and chitosan [50]; however, the fabrication of aligned fibers loaded with this drug appears to be unpublished. Aligned fiber morphology encourages directional growth in neurons and Schwann cells, and has been demonstrated to improve regeneration in animal models [32–34], and so is desirable in biomaterials for nerve repair. Our previous work used tacrolimus-loaded PCL fibers for the local immunosuppression of nerve allografts [37], whereas in this study we have adapted that approach to explore the delivery of this neurotrophic drug in combination with aligned topography in order to support axonal regeneration directly. The physiochemical and functional properties of the fibers were characterized, alongside their efficacy as scaffolds for Schwann cells and primary rat neurons. The materials demonstrated controlled release of tacrolimus up to 1 month, alongside positive modulation of genes related to regeneration and inflammation in Schwann cells. Primary rat neurons from dorsal root ganglia displayed robust and orientated outgrowth of neurites on the aligned drug-loaded fibers, further demonstrating their potential as scaffolds for nerve regeneration.

The electrospinning fabrication route generated materials with fibers of consistent diameter, and high mandrel collector speeds effectively aligned the blend tacrolimus-loaded PCL fibers. The aligned fibers generated here had a diameter approaching half of that of the randomly orientated materials, which may have resulted from mechanical stretching of the fiber onto the fast-moving collector [51,52] alongside a lower initial polymer concentration. Better regeneration outcomes have been achieved using 250 nm aligned PCL fibers compared with 980 nm in a rat sciatic nerve injury model [53], so this reduction in fiber diameter may be beneficial. The tacrolimus-loaded random fibers were characterized by XRD; however, only peaks typical of PCL were evident. This could suggest that tacrolimus was encapsulated in an amorphous state – a well-established effect in electrospinning as the fast evaporation of solvent can prevent recrystallization [54]; however, the low mass of drug loaded into the fibers may also have been below the detection limit of XRD, which is reported as a few percent crystallinity [55]. FTIR analysis of the drug-loaded materials was also virtually identical to the blank fibers, which again may be expected given the small amount of tacrolimus encapsulated. As a result, these analyses were not performed for aligned fibers.

The duration of tacrolimus release from random and aligned materials was measured at 32 and 26 days, respectively – this timeframe is in line with the release profiles of tacrolimus-loaded materials that have been efficacious in rodent models [17–20]. Notably, the aligned fibers exhibited a higher proportion of burst release than the random fibers. This is likely to be a result of the blend electrospinning method that was used to make the aligned fibers compared with the coaxial electrospinning method used for the random fibers. Blend fibers usually display more rapid release of therapeutics compared with those generated by a coaxial process, as demonstrated in cefazolin-loaded PCL fibers [56], as the drug is dispersed randomly throughout the polymer matrix and that close to the surface rapidly diffuses out when placed in solution.

LDH activity in the media of Schwann cells cultured on the electrospun fibers was measured after 48 h in order to assess the ability of the scaffolds to support the adhesion and growth of a cell type relevant to nerve regeneration. LDH is released from the damaged plasma membrane of dying cells, and its activity can be assayed through enzymatic production of chromogenic formazan [57]. In both random and aligned conditions the blank and drug-loaded electrospun fiber groups had similar levels of LDH activity, indicating that the released tacrolimus did not exert a cytotoxic effect, and cell death above that on TCP was minimal.

The effect of both nanofibrous surface topography and the presence of tacrolimus on gene expression in these Schwann cells was then elucidated using RT-qPCR, with a focus on a panel of nine genes involved in regeneration or inflammation. In random materials, seven out of nine genes were differentially expressed on blank fibers compared with TCP, illustrating the significance of topography in regulation, and four genes were also modulated by exposure to tacrolimus from the drug-eluting fibers. In particular, although *Bdnf* expression was highest on TCP, it was positively regulated on tacrolimus-loaded fibers compared with blank. BDNF is a survival factor for motor neurons [58] and is critical for nerve regeneration after injury [59]. Local BDNF delivery from microspheres [60] or in the form of peptide mimetics [61,62] promotes regeneration in rat models, and so upregulation of this gene and in turn its protein product may be beneficial for nerve repair. BDNF and its receptor TrkB were also increased in the spinal cord motor neurons of nerve-injured rats treated with a tacrolimus-loaded conduit [63], so the positive regulation found in this study appears to have precedent. In contrast, the expression of *Lif* was markedly reduced in cells cultured on electrospun fibers. Suppression of this factor has been found to enhance Schwann cell migration and proliferation and improve outcomes in a rat model of nerve injury [64], and so this inhibition may be beneficial for regeneration. In addition, the expression of *Sox10* was reduced on random blank fibers compared with TCP, and further reduced on drug-loaded fibers compared with blank. This factor is involved in regulating the expression of myelinating genes, and reduced expression of pro-myelinating factors has also been reported in primary rat Schwann cells seeded on aligned poly(lactic-co-glycolic acid) fibers, when materials contained the immunomodulatory drug fingolimod [65].

These effects were not always replicated in aligned materials, where *Bdnf* and *Sox10* expression were similar in the presence and absence of tacrolimus. This differential between aligned and random materials may be a function of tacrolimus dosage or different electrospinning methods: as aligned scaffolds were collected on a larger mandrel in order to produce sufficient surface velocity to align the fibers, they were thinner than random materials and contained less drug per area. The expression of *Il6* on random and aligned fibers was also dissimilar, where random blank and tacrolimus-loaded fibers promoted significant upregulation compared with TCP and those of aligned fibers had no differential effect. In preclinical nerve regeneration research, IL-6 is regarded as a proinflammatory cytokine – for example, primary rabbit Schwann cells [66] and RT4 Schwann cells [67] stimulated with the endotoxin lipopolysaccharide exhibited increased expression of this gene. As such, that aligned fibers did not incite this response may be favorable if employed as a biomaterial for nerve repair. Similarly, while the expression of *Jun* was negatively modulated on random drug-loaded fibers compared with TCP, there was no such effect on the aligned counterpart. Schwann cells respond to stiffer substrates in part through downregulation of *Jun* [68]; however, as aligned PCL fibers are reported to be stiffer than random fibers [69], that may not account for this observation. Given the critical role of c-Jun in nerve regeneration, that tacrolimus and aligned nanofibrous topography do not impact its expression compared with TCP may be a valuable finding in the design of scaffolds for nerve repair. Conversely, the downregulation of *Lif* observed on random fibers was echoed on aligned fibers – as aforementioned, this effect may be advantageous as suppression of LIF has been shown to promote regeneration in rodents [64]. Future studies should investigate whether the differential expression of pro-regenerative and inflammation-related genes found here translates into changes in the level of these proteins.

The morphology of Schwann cells cultured on the drug-loaded aligned fibers was also investigated and compared with that on aligned and random blank fibers without tacrolimus. The directional scaffolds encouraged the cells to align, an effect that has been reported previously for aligned PCL fibers in primary human fetal Schwann cells [29], primary rat adult Schwann cells [24] and the RSC96 Schwann cell line [70]. The presence of tacrolimus was not detrimental to this orientation effect. The alignment and elongation of Schwann cells in culture is reminiscent of bands of Büngner, which are the structures formed by repair-phenotype Schwann cells after nerve injury that guide the migration of axonal growth cones [71]. Emulating these structures by implanting aligned Schwann cell-laden constructs has been found to promote regeneration [72], and so the ability of the aligned drug-loaded fibers to incite this morphology may enhance *in vivo* efficacy.

Finally, the ability of the scaffolds to direct the outgrowth of neuronal cells was evaluated using cultures of primary rat sensory neurons. Compared with random fibers, the drug-loaded and blank-aligned fibers encouraged greater and more directional outgrowth, although the length of the neural clusters was not increased by the presence of tacrolimus. Tacrolimus has been shown to promote neurite outgrowth *in vitro* in primary embryonic explant cultures [6,18,19,73,74], and so an increase in length was anticipated. This discrepancy may be explained by the narrow therapeutic window of tacrolimus – in dissociated embryonic chick DRG cultures, 1 μM tacrolimus (804 ng/ml) was found to inhibit outgrowth compared with controls [75], which is in line with the approximately 800 ng/ml of tacrolimus released from the aligned fibers at 48 h. As such, future work could involve a reduction in loading dose in order to produce an efficacious concentration for neurite outgrowth in the local delivery area. Furthermore, future investigations to assess the efficacy of the orientated drug-loaded biomaterial *in vivo* would be highly valuable, as this more complex environment includes cell types such as macrophages, which are known to positively influence regeneration using aligned fibers [35,76].

## Conclusion

This study reported the fabrication and characterization of random and aligned electrospun biomaterials with sustained release of tacrolimus over multiple weeks. Changes in gene expression in Schwann cells were observed in response to both random and aligned nanofibrous topography, in addition to the presence of tacrolimus. Schwann cells and primary rat neurons cultured on the aligned materials exhibited robust and orientated growth, demonstrating their potential for use in nerve tissue engineering.

**Summary points**

- Severe peripheral nerve injury commonly requires microsurgical repair, but these gold standard treatments may not effectuate the return of sensory/motor function.
- Biomaterials with encapsulated neurotrophic drugs such as tacrolimus implanted at the injury site could enhance regeneration.
- Electrospun poly-ε-caprolactone scaffolds with random and aligned fibers containing tacrolimus were generated and characterized.
- Gene expression profile of Schwann cells cultured on the biomaterials was influenced by nanofibrous topography and presence of tacrolimus.
- Schwann cells on aligned fibers displayed an aligned and elongated morphology akin to bands of Büngner.
- Aligned fibers supported and directed robust outgrowth from primary rat neurons.

## Open access

This work is licensed under the Creative Commons Attribution 4.0 License. To view a copy of this license, visit http://creativecommons.org/licenses/by/4.0/
